# Behavior Modification Maintenance with Long-Term Blood Glucose and Weight Management in Prader–Willi Syndrome Complicated with Diabetes: Team Management Approach Combined with Pharmacological Treatment

**DOI:** 10.1155/2019/6129019

**Published:** 2019-07-08

**Authors:** Eri Kato, Moritsugu Kimura, Tomoyuki Okuda, Masao Toyoda, Masafumi Fukagawa

**Affiliations:** ^1^Seichi Clinic, Isehara, Kanagawa, Japan; ^2^Division of Nephrology, Endocrinology and Metabolism, Department of Internal Medicine, Tokai University School of Medicine, Isehara, Kanagawa, Japan

## Abstract

The patient was a 40-year-old woman, who had been diagnosed with Prader–Willi syndrome (PWS) at 1 year of age and type 2 diabetes at 27 years of age. At 34 years of age, she was hospitalized to start insulin therapy and receive guidance on treatment. During the next 6 months and through regular once-monthly outpatient clinic visits, the blood glucose level was relatively stabilized although body weight gradually increased. Two years following discharge, the blood glucose level became unstable, and she was hospitalized again to receive guidance on treatment. A team medicine-based approach was established upon hospitalization. The basic treatment was unchanged (insulin, diet, and exercise). The approach taken by the team included understanding the characteristics of PWS by all team members, clear definition of treatment goals, positive evaluation of the patient, and maintenance of the patient's motivation for treatment. Anxiety and stress related to mother's illness dampened motivation and adherence to treatment, but the addition of appropriate pharmacological treatment helped in rapid recovery of motivation to adhere to the treatment protocol. At 3 years after discharge, HbA1c is maintained at around 6%, and body weight continues to fall. Our protocol of the combination of a team medicine approach with appropriately timed pharmacological intervention could probably be applied to not only type 2 diabetes in PWS but also the management of patients with poorly controlled type 2 diabetes.

## 1. Introduction

Prader–Willi syndrome (PWS) is one of the most common genetic obesity syndromes caused by lack of expression of genes on the paternally inherited chromosome 15q11.2-q13 region. There are three main genetic subtypes in PWS: paternal 15q11-q13 deletion (65–75% of cases), maternal uniparental disomy 15 (20–30% of cases), and imprinting defect (1–3%). The condition is characterized by intellectual impairment and gross obesity associated with overeating [1, 2]. PWS is often complicated with type 2 diabetes mellitus [3], and some studies reported difficulties in the overall control of body weight and blood glucose levels over the long term in PWS patients [4]. However, a few reports have described the achievement of good results with home treatment [4–6]. To manage the weight gain and blood glucose level instability that characterizes PWS, conventional treatment has been implemented in the hope of eliciting behavior modification. Such behavior modification ultimately reaches the maintenance phase (stage 5) after the precontemplation phase (stage 1) and contemplation phase (stage 2). However, behavioral problems due to explosive emotions and persecutory delusions tend to perpetuate a cycle of behavior modification failure and regression.

We report here a rare case of PWS in which favorable weight and blood glucose level management could be maintained over the long term with home care by the combination of thorough and positive team medicine coupled with appropriate pharmacological intervention despite typical difficulties in the management of body weight and blood glucose levels.

## 2. Case Presentation

The patient was a 40-year-old woman who presented with a chief complaint of weight gain. She had a family history of type 2 diabetes in both parents.

### 2.1. History of Current Illness

The patient was diagnosed with PWS at the age of 1.5 years based on genetic testing performed at a pediatric hospital. After the diagnosis, the patient attended regular health checkups and was diagnosed with type 2 diabetes by the local internist at the age of 27 years. That year, the patient was referred to a teaching hospital to undergo treatment for diabetes, where she started to attend regular outpatient examinations. She was placed on dietary and exercise therapy only, but at the age of 28 years, she was prescribed an alpha-glucosidase inhibitor based on HbA1c of 7.8%. Oral glucose-lowering agents, including sulfonylureas, were later added and doses amplified, but the HbA1c level did not stabilize, varying from around 7 to 9%. Insulin therapy was started at the age of 34 years, and the patient was admitted to our clinic in 2013 to undergo guidance for this treatment. At admission, HbA1c was 9.4% and body weight 80 kg. She underwent the routine diabetes guidance educational program offered at our clinic and was discharged 2 weeks later.

After discharge, she attended regular outpatient examinations once per month and also continued to undergo frequent injection therapy. Six months later, while HbA1c had improved from 9.4% to 7.2%, body weight increased to 85.4 kg. Subsequent dietary and exercise therapy offered on an outpatient basis was ineffective. In 2015, at the age of 36 years, HbA1c increased to 8.3%, and she was hospitalized again for management of the blood glucose level and to receive further guidance and education.

### 2.2. Intellectual Level

The patient was able to read and write simple phrases and ranked 15/30 on the Revised Hasegawa's Dementia Scale (HDS-R) and 18/30 on the Mini-Mental State Examination (MMSE). The patient refused to undertake the Wechsler Adult Intelligence Scale (WAIS).

### 2.3. Character

The patient disliked complex expressions and actions, being preached, and getting into disputes. She preferred simple expressions and actions and being praised and evaluated and liked bright atmosphere.

At the time of hospitalization in 2015, height was 152 cm (average height of age-matched Japanese women: 158 cm), weight 85 kg, BMI 36.8 kg/m^2^, thick subcutaneous fat, low muscle mass, HbA1c 8.3%, arterial blood pressure 145/70 mmHg, pulse rate 78 bpm (regular), body temperature 35.5°C, and consciousness lucid.

### 2.4. Oral Medications

Insulin aspart (12-7-12 units), insulin degludec (21-0-0 units), metformin hydrochloride (750 mg/day), and rosuvastatin calcium (2.5 mg) were given. She was on 1200 kcal/day and 6 g/day of salt [7]. Laboratory tests at hospitalization (Table 1) showed fasting blood glucose of 194 mg/dl and HbA1c of 8.3%, indicating poor blood glucose control. Investigation of diabetes-related complications identified phase 1 nephropathy, but neither diabetic retinopathy nor neuropathy.

### 2.5. Course following Hospitalization

Consultation with medical staff of different specialties on the potential cause(s) of the patient's inability to manage body weight and blood glucose levels over the long term identified several possible causes. These included the fact that the regular inpatient guidance program was implemented without any understanding of the patient's background, including the characteristics of PWS; lack of smooth coordination between the patient, family, and worksite and medical staff; and difficulty in maintaining motivation among the medical staff for time- and energy-consuming treatment. The patient was also noted to engage in secret snacking, and when reprimanded for this, she started reporting false blood glucose levels. Therefore, during the second hospitalization in 2015, more understanding of the patient's PWS was offered. Care was taken not to reprimand the patient for snacking or similar behavior, and she was praised for honestly reporting blood glucose levels, allowing her to experience a feeling of success in being able to manage own diet, and care was taken to engage with the patient based on the goal of enhancing motivation for treatment. Efforts were made to engage in smooth coordination to confirm the accuracy of the patient's blood glucose and dietary records by having the patient, her family, worksite staff, and medical staff regularly confirm and evaluate the credibility of her records. Care was also taken to maintain the motivation of the medical staff involved in long-term treatment by making them aware that the experience gained in the treatment of this patient would form valuable assets for their future medical career. The medical staff involved in the treatment included a physician, nutritionist, physical therapist, nurse, and clinical technologist. As we do not have pharmacists at our clinic, the physician also took on the role of the pharmacist. Since treatment with glucose-lowering agents, including insulin, was confirmed to be appropriate from before hospitalization, treatment during hospitalization was focused on diet and exercise therapy.

**Figure 1:**
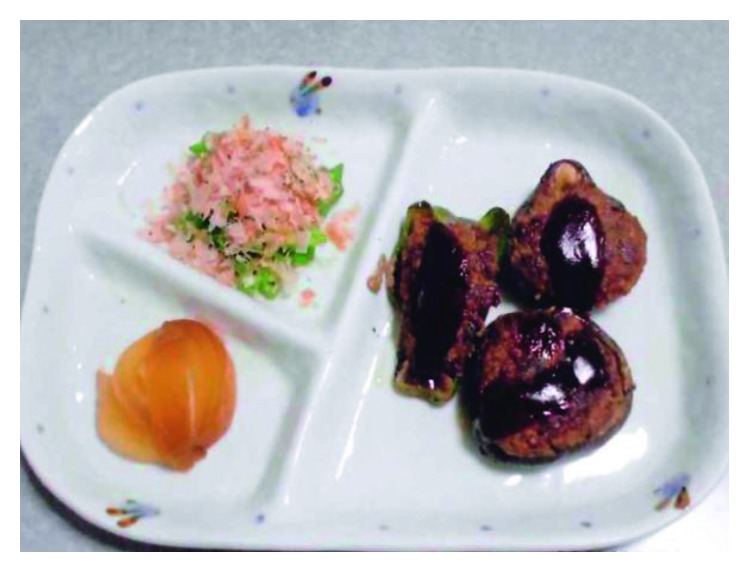
The main portion of one meal.

**Figure 2:**
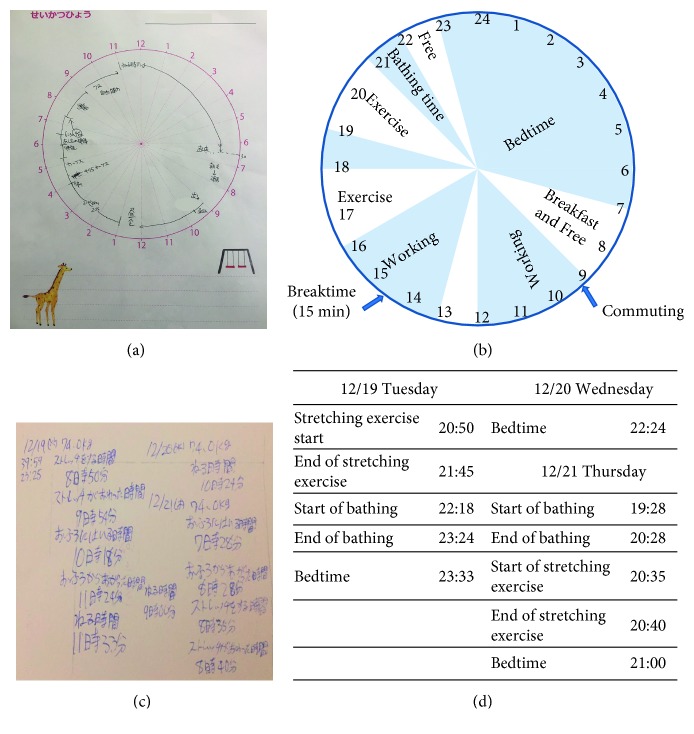
Examples of actual daily schedule ((a) Japanese; (b) English) and time of exercise ((c) Japanese; (d) English) used to evaluate changes in the patient's daily lifestyle and treatment behavior following discharge. The patient submitted these at her monthly examinations, and information such as specific exercise time was used for team evaluation.

Since the understanding and cooperation of family members and persons close to the patient are essential for dietary therapy [8], the nutritionist collaborated with the family and worksite staff while offering nutritional guidance to the patient [9]. The nutritionist then established the goals of treatment and reported on the details of the guidance offered in the presence of family members. The family was asked to avoid providing buffet-style meals, but to serve the meals on a single plate taking size into account (Figure 1) [10]. Staple food at amounts equal to those offered at home was also provided for meals taken at the patient's worksite, and the patient was requested to convey these changes to the worksite staff. Since establishing fun and rich mealtimes that are a happy family get together are recommended for reducing mental stress [11], the family was asked to serve meals for all family members, including the patient. Repeatedly offering individually tailored dietary guidance, while taking the patient's psychological age, intelligence quotient, and mental status into account, is considered to be important when administering dietary therapy to diabetes patients with intellectual disabilities [11–15]. As the intellectual level of our patient meant that she could read and write simple phrases, we considered that she could understand her daily directed nutrient intake. Therefore, we proposed and helped the patient select dietary therapy options that would not place too heavy a burden on her and would enable her to continue with the therapy. Furthermore, guidance on intake methods that matched specific situations, such as purchasing drinking water from a vending machine or during events, such as birthday parties, is also important for PWS patients [10], such explanations were repeatedly offered in a careful and easy-to-understand manner. The daily food intake was recorded by photographs and daily schedules (Figures 1 and 2), from which the daily energy intake was calculated.

At the time of hospitalization, height was 152 cm, weight 85 kg, and BMI 36.8 kg/m^2^. The height was slightly lower than the average height of 158 cm for Japanese women of the same age, advanced obesity was recognized, subcutaneous fat was thick, and the muscle mass was low, which were consistent with the characteristics of PWS [2]. Although we could not evaluate insulin resistance due to the complex setup requirements and the patient character, we considered the patients to have high insulin resistance based on the continuous need for insulin therapy after the correction of glucose toxicity. Furthermore, it was also assumed that insulin secretion was intact based on the 24-hour urinary C-peptide level of 67.9 *μ*g/day at admission. Considered together, the patient started exercise therapy supervised by a physiotherapist while she was treated with metformin (insulin-sensitizing drug).

**Figure 3:**
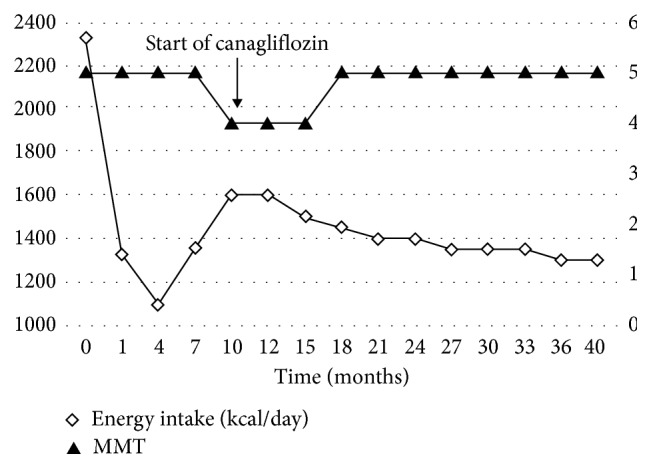
Energy intake recorded by the patient during 40 months after the second hospitalization for treatment guidance, plotted with the results of manual muscle testing (MMT). Changes in the home environment (mother illness) at 5 months after discharge were associated with increased daily energy intake and decrease in quadriceps femoris muscle MMT. Canagliflozin was subsequently started at 10 months after discharge in order to deal with these changes and correct blood glucose levels.

In our case, exercise therapy was tailored to the clinical features of PWS, taking into account the significant decrease in physical activity associated with obesity, insomnia, and muscle weakness. Furthermore, such therapy was based on the concept that physical exercise and exercise programs are beneficial in improving body composition [16–19]. While the patient waited to undergo medical examination, the physical therapist confirmed the patient's lifestyle rhythm (Figures 2(a) and 2(b)) and details of the behavior records (Figures 2(c) and 2(d)) over the preceding month. In order to select a proper exercise protocol, the patient also underwent muscle mass assessment and manual muscle testing (MMT) for the quadriceps femoris muscle. Furthermore, the selected exercise load was applied to improve body composition and enhance the sleep quality (Figure 3).

The nurse and clinical technologist constantly provided care with an empathetic attitude [20]. They offered care, such as checking the patient's insulin injection technique and medication status, and also implemented foot care. Furthermore, the staff at the patient's worksite regularly visited our clinic and shared information with the medical staff while providing updates on the patient's recent status at worksite and home.

**Figure 4:**
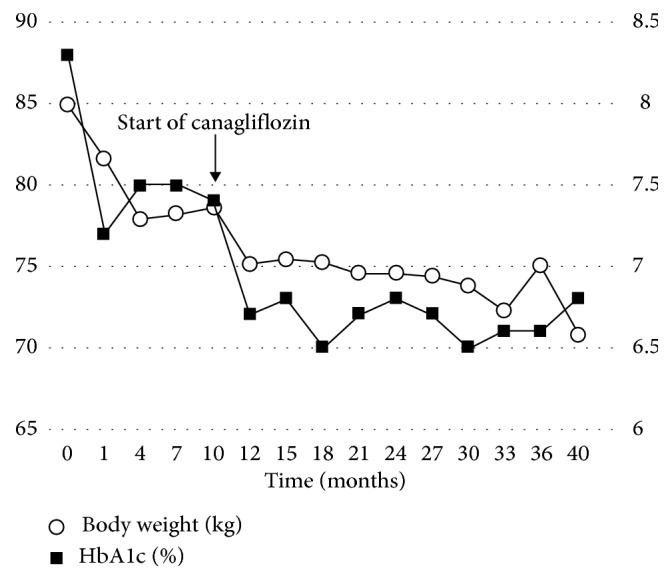
Changes in HbA1c and body weight during the 40 months after the second hospitalization, plotted for treatment guidance. Canagliflozin administration was started at 10 months after discharge in response to changes in energy intake.

Careful observation of these practices resulted in improvement of HbA1c level from 8.3% to 7.5% and decrease in body weight from 85 kg to 78 kg within 5 months of hospital discharge (Figure 4). However, at 10 months after discharge, the patient apparently stopped observing the daily energy intake and exercise load (Figure 3). Investigation by the team members found that the cause was a major change in the patient's home environment, with chronic condition, multiple myeloma, affecting her mother for a long time, who had been the closest and most involved individual in the daily care of our patient. This made it difficult for the patient to maintain motivation for treatment. At the evaluation meeting held by the team, it was confirmed that the patient's daily energy intake had increased, and MMT had decreased to 4, while the daily schedule records showed she had completely ignored dietary and exercise therapy requirements, which was attributed mainly to the loss of motivation. Since this loss of motivation was related to anxiety about her mother, the management team members selected a multidisciplinary approach to increase the patient's sense of self-responsibility by allowing her to experience success in order to regain motivation. Specifically, this experience of success reflected the improvement in HbA1c levels and body weight. Accordingly, canagliflozin, a glucose-lowering SGLT2 inhibitor, was added to the treatment at 100 mg/day. The addition of canagliflozin improved not only HbA1c and body weight but also daily energy intake, a change that could not be attributed to the action of the pharmaceutical agent. In response to this, the MMT results also recovered to the level prior to drug administration, and she started engaging in more physical exercise. This was confirmed through evaluation of physical strength required for daily lifestyle, such as gait analysis and stability during outdoor walking, stair climbing and descending speed, and a decrease in required nursing care (Figures 3 and 4). The patient continued to manage her blood glucose level and body weight well. At the last clinical checkup, 3 years after discharge from the hospital (June 2018), diabetes was under control with insulin aspart (6-0-8 units), insulin degludec (12-0-0 units), metformin (750 mg/day), and canagliflozin (100 mg/day). HbA1c was at 6.8%, and body weight was 70.8 kg (Figure 4).

## 3. Discussion

PWS is associated with intellectual impairment and gross obesity due to overeating. Overeating from infancy progresses to gross obesity from school years through to adulthood, ultimately leading to diabetes [4]. Several studies reported difficulties in the management of body weight and blood glucose in diabetic PWS patients [4–6, 21, 22]. Overeating in PWS is considered to be pathologically caused by hypothalamic impairment that disrupts the normal feedback mechanism of feeling of fullness and decreased hunger after eating [23, 24]. One study also reported the utility of using GLP1 receptor agonists for PWS [5]. While our patient was prescribed GLP1 receptor agonists, they were discontinued after a short period of time due to lack of clear clinical response. GLP1 receptor agonists are thought to act in PWS as appetite inhibitors through the suppression of ghrelin secretion [25]. Previous studies showed that the addition of exenatide in diabetic PWS patients allowed the reduction of insulin dosage, but such effect disappeared after 6 months of treatment [26]. Based on our experience, we believe that the limited effect could be related to the difficulty in long-term maintenance of body weight and blood glucose level by pharmaceutical intervention alone. Since we felt that continuation and intensification of behavior modification through a team medicine-based approach could act synergistically with pharmacotherapy, we implemented the following actions.

Previous studies stressed the importance of tackling problems of PWS in coordination with the patients, their families, and medical staff [20, 21, 27]. Therefore, during the second hospitalization, we engaged in coordination between the patient, her family, and worksite staff, after ensuring all medical staff sufficiently understood and shared information on PWS characteristics and the patient's mental status and personality, to tackle the problem of overeating and frequent snacking.

It is important to respect the wishes and psychological characteristics of PWS patients, so as to help them feel satisfied, bestow a sense of pride, and achieve the desired goals [28–30]. It has been reported that the medical staff should sensitively respond to the patient's behavior modification, rejoice with them, and praise them and that repeating this cycle can encourage the habit of behavior, resulting in treatment efficacy [7, 22]. Therefore, in contrast to the vague goals set upon initial hospitalization, we setup two clear goals, i.e., weight loss and stabilization of blood glucose levels, in the second hospitalization. Setting these clear goals also made it easier for the medical staff, the patient herself, the family members, and the worksite staff to assess the patient's progress and increased the number of opportunities for interdisciplinary communication. Assessment was conducted by evaluations at each regular outpatient examination. If the patient displayed even a minute positive attitude toward treatment, this was judged to indicate a common recognition of positive evaluation. On the other hand, when inappropriate behavior was noted, the staff empathized with the patient's emotions rather than pointing out the inappropriate behavior, and she was provided with repeated, easy-to-understand explanations on appropriate behavior and sufficient praise when she improved her behavior. In this regard, Haig and Woodcock advised that even if the patient engaged in repeated inappropriate behavior, the staff should take care to continuously display a unified stance [31].

It has been reported also that PWS patients may engage in behavior such as deliberately reporting erroneous information [32]. As our patient was also noted to engage in similar behaviors at times, we took care from the second hospitalization onward to encourage smooth coordination between the patient, family, worksite staff, and medical staff, including confirming whether erroneous information had been recorded. When it became difficult for the patient's mother to oversee the patient's care, closer coordination was implemented with the patient's father and worksite staff, including regular confirmation and evaluation of the credibility of information reported by the patient. We also matched photographs of meals with information recorded in the patient's daily schedule.

It is desirable to use body composition analysis equipment or similar means to evaluate the effects of diet and exercise therapy conducted by a professional medical team. However, since no such equipment was available at our clinic, evaluation was limited to daily energy intake, quadriceps femoris muscle MMT, HbA1c, and body weight. We estimated the daily energy intake based on meal details and meal photographs recorded by the patient (Figures 1 and 2). While the estimated prehospitalization energy intake was approximately 2300 kcal, it decreased to roughly 1200–1400 kcal at 5 months after discharge while the patient had maintained MMT of 5. Consequently, the patient lost weight gradually, and this was accompanied by a fall in HbA1c level. However, from 5 months after discharge, a major change in the patient's home environment related to her mother's health resulted in lack of adherence to the management protocol, including increased energy intake and a fall in MMT to 4 (Figure 3). It should be noted that the reproducibility of MMT is questionable, and physical strength in this study was assessed subjectively by a single physical therapist. Such evaluation might have lacked reproducibility and objectivity. Further multicenter studies are needed in which more reproducible and objective evaluation methods are used.

PWS patients are known to be sensitive to stress and can be affected easily by mood disorders [7, 33, 34]. The serious illness of the patient's mother, who lived and looked after the patient, resulted in a significant change to the patient's home environment, thus subjecting the patient to psychological stress. Therefore, the management team elected to implement the characteristics of PWS into account, as well as the patient own recent success as an opportunity to help the patient recover motivation and increase the sense of self-efficacy. As the patient regressed from maintenance out of the five stages of behavior modification (precontemplation, contemplation, preparation, action, and maintenance) [35], the team discussed the type of pharmaceutical intervention that could achieve the required effects in an appropriate and timely manner. The plan behind the use of the SGLT2 inhibitor was to evaluate the expected improvements in the HbA1c level and body weight and then use these results to increase patient's motivation. After the start of canagliflozin, the patient exhibited improvements in both HbA1c and body weight despite her mother's health status. While these results may first appear as simply resulting from the administration of canagliflozin, we were able to confirm decreased daily energy intake and engagement in physical exercise, both of which could not be the effects of the pharmaceutical agent alone. We believe that team medicine utilizing the effects of pharmacological intervention played an important role in the long-term improvement in the HbA1c level and weight loss. However, the methods of evaluation used in our case had some limitations in terms of reproducibility and objectivity. As our evidence may have had somewhat weak scientific and objective grounds, a more objective means of evaluation of PWS needs to be developed.

Our experience in the treatment of this patient demonstrated that implementation of treatment of diabetes in PWS patients requires the following actions. Medical staff should work in a multidisciplinary manner to grasp the characteristics of PWS, clearly define treatment goals, positively evaluate the patient, and maintain motivation for treatment with a good relationship of trust between the patients, their family, and individuals involved in the treatment. In addition, if the patient is found to exhibit regression of behavior modification, appropriate and timely pharmacological intervention appears to be important to improve and subsequently maintain motivation. The methods identified through the treatment of our patient (diet and exercise therapy) could be also utilized in the treatment of patients with poorly controlled diabetes and obesity. Accumulation of more data on such a treatment approach should help refine the management plan of PWS patients.

## Figures and Tables

**Table 1:** Results of laboratory tests.

*Urinalysis*	
pH	7.0
Protein	(−)
Glucose	(−)
Ketone	(−)
Blood	(±)
Specific gravity	1.010
24-hour urinary C-peptide	67.9 *μ*g/day

*Blood cell counts*	
Leukocyte count	7.96 × 10^3^/*μ*l
Erythrocyte count	5.08 × 10^6^/*μ*l
Hemoglobin	14.8 g/dl
Hematocrit	45.7%
Platelet count	35.6 × 10^6^/*μ*l

*Blood chemistry*	
Glucose	194 mg/dl
HbA1c	8.3%
Total protein	7.6 g/dl
Albumin	4.6 g/dl
BUN	11.8 mg/dl
Creatine	0.14 mg/dl
Uric acid	4.5 mg/dl
Na	139 mEq/l
K	4.3 mEq/l
Cl	97 mEq/l
AST	18 U/l
ALT	20 U/l
Total cholesterol	229 mg/dl
HDL-C	60 mg/dl
LDL-C	158 mg/dl
Triglycerides	172 mg/dl

BUN = blood urea nitrogen; AST =  aspartate aminotransferase; ALT =  alanine aminotransferase; LDH = lactate dehydrogenase; ALP = alkaline phosphatase; HDL-C = high-density lipoprotein-cholesterol; LDL-C = low-density lipoprotein-cholesterol.
